# Supplementary material for: MetaRibo-Seq measures translation in microbiomes
Source: Nat Commun. 2020 Jun 29;11:3268. doi: 10.1038/s41467-020-17081-z (PMC7324362; doi:10.1038/s41467-020-17081-z)
Supplement: Supplementary file 10 — Supplementary Data 7 [file 41467_2020_17081_MOESM10_ESM.zip › File2/Confidence_High_Taxonomy/169011_out.krona.html]

Javascript must be enabled to view this page.

members
magnitude
magnitudeUnassigned
count
unassigned
taxon
rank

169011\_out

2

superkingdom
2
2

1239
2
phylum

186801
2
class

2
186802
order

2
541000
family

genus
2
1263

species

SRS078176\_contig\_number\_9892
1
1262959

1
1262961
species

SRS147557\_contig\_number\_9032
